# Diagnosis of Congenital Cytomegalovirus Infection in High Risk Neonates

**DOI:** 10.4084/MJHID.2013.049

**Published:** 2013-07-10

**Authors:** Ehab Abd Elmoniem Albanna, Randa Saddek Abd El-latif, Hend Alsayed Sharaf, Maha Kamal Gohar, Basem Mohamed Ibrahim

**Affiliations:** 1 Pediatric, Faculty of Medicine, Zagazig University.; 2 Microbiology & Immunology, Faculty of Medicine, Zagazig University.; 3 Ophthalmology Departments, Faculty of Medicine, Zagazig University.

## Abstract

**Objectives:**

This study aimed to compare polymerase chain reaction (PCR) and IgM detection using enzyme linked immune-sorbent assay (ELISA) in diagnosis of congenital cytomegalovirus (CMV) infection.

**Methods:**

This study was conducted from May 2009 to December 2010. Urine and blood samples were collected from 94 neonates with suspected congenital CMV infection. Serum and part of urine samples were stored at −20°C freezer, until the serologic and PCR tests were achieved. A 94 fresh urine samples were processed for cell culture. Nineteen (20.2%) out of 94 urine samples were proven positive for CMV infection by viral culture. For comparing PCR and IgM ELISA we used tissue culture technique as a reference, the 19 positive samples on culture (CMV group) and 20 negative samples (control group) were included in the comparison. Some characteristics of CMV and control groups were compared including sex, age, birth weight, gestational age < 37 and small for gestational age. Clinical and laboratory abnormalities were also compared in both groups.

**Results:**

This study showed that the sensitivity and specificity of PCR in relation to viral culture were 100% and 100% respectively, there was excellent agreement between both tests (Kappa coefficient was 1 and P=0.000). On the other hand, the sensitivity of IgM CMV ELISA in relation to viral culture was 63.2% and the specificity was 85%. There was good agreement between both tests (Kappa coefficient was 0.48 and P=0.002). By comparing CMV and control groups, there were high statistically significant differences between both groups as regard the birth weight, gestational age < 37 and small for gestational age items (P= 0.00, 0.03 and 0.01 respectively). There were statistically insignificant differences as regarding the clinical and laboratory abnormalities detected for neonates of both groups. In this study jaundice (63%) and hepato-splenomegaly (42%) were the most common clinical signs in both groups.

**Conclusions:**

PCR is more sensitive and specific technique for detection of congenital CMV infection than CMV IgM ELISA. Being more cost effective, less cumbersome and less time consuming in relation to viral culture, PCR may be used in detection of congenital CMV infection.

## Introduction

Cytomegalovirus is a species of virus that belongs to the Beta-herpesvirinae subfamily which is a part of a viral family known as Herpesviridae or herpesviruses.^1^ Although they may be found throughout the body, CMV infections are frequently associated with the salivary glands.^2^ Cytomegalovirus infection is typically unnoticed in healthy people, but can be life-threatening in immunocompromised, such as HIV-infected persons, organ transplant recipients, or new born infants. CMV infection has an ability to remain latent within the body for long periods. 1

Cytomegalovirus is also the virus most frequently transmitted to a developing fetus. Cytomegalovirus infection is more widespread in developing countries and in communities with lower socioeconomic status and represents the most significant viral cause of birth defects in industrialized countries. 3

The risk of intrauterine transmission after primary CMV infection during pregnancy approaches 40%, with an increased risk of adverse fetal effects if infection occurs during the first half of pregnancy^4^. Of congenitally infected infants, approximately 10% are symptomatic at birth. Of the remaining 90% of infants who are asymptomatic at birth, 10%–15% will subsequently manifest evidence of permanent sequelae.^5^

Typical clinical symptoms of congenital CMV infection that are found in infected neonates include intrauterine growth retardation, microcephaly with intracranial calcification, hepatosplenomegaly, jaundice, chorioretinitis, thrombocytopenic purpura and anemia.^6^

Rapid and correct diagnosis of congenital CMV infection in neonates is very important to advocate the right therapy and proper management of the case. ELISA was previously used for detection of CMV specific IgM class antibodies to establish current or congenital CMV infection but low specificity and sensitivity of the ELISA systems used have been reported in some evaluation studies.^7^

Cytomegalovirus affected babies are known to shed virus in various body secretions especially urine, blood and throat swab, in some cases, for months and years. Detection of cytomegalovirus in clinical samples like urine and blood, by PCR, also provides important information about the excretion of virus in the infected baby and prediction of the symptomatic disease.^8^

The objective of the present study was to compare PCR and IgM ELISA in diagnosis of congenital CMV infection.

## Materials and Methods

This study was conducted at Pediatric and Microbiology & Immunology departments, Faculty of Medicine Zagazig University hospitals from May 2009 to December 2010.

### Patient criteria

Ninety four neonates suspected of congenital CMV infection were included in this study. 88 neonates with clinical symptoms compatible with congenital CMV and 6 born to mothers with a history of primary CMV infection during pregnancy. All cases were less than 21 days in age to distinguish congenital infection from the more common, but clinically benign, perinatal infection. 9 For each patient data were collected including age, sex, birth weight, gestational age and mother’s history of CMV infection during pregnancy. Clinical signs and symptoms of congenital CMV infection were registered including microcephaly (small head size), small for gestational age (small body size), petechiae (little red spots under the skin), purpura (larger purple spots under the skin), hepatosplenomegaly, jaundice, seizures, , chorioretinitis (inflammation of the back of the eye that can cause blindness), and deafness. Neonates may have one or more than one of these signs or symptoms. 8 Complete clinical examination of all neonates was done by the pediatrician. Laboratory findings were registered for each neonate including CBC and Liver function tests including bilirubin. X-ray of the chest was also done.

Fundus examination was done using indirect ophthalmoscope for the all 94 high-risk cases for CMV infection; pupillary dilatation was achieved by 3 times installation (10 minutes apart) of a combination of cyclopentolate 0.2% and phenylephrine 1%.

This combination was prepared by mixing equal amounts of diluted cyclopentolate 0.4% and diluted phenylephrine 2%, diluted cyclopentolate 0.4% was prepared by addition of 1.5 ml BSS (Balanced Salt Solution) to every 1 ml of commercially available cyclopentolate 1% (cicloplejico 1% Alcon USA) while diluted phenylephrine 2% was prepared by addition of 0.25 ml BSS to every 1 ml of commercially available phenylephrine 2.5% (phenylephrine 2.5% Misr co. Egypt).

### Sample collection

Urine and blood samples were collected from 94 neonates with suspected congenital CMV infection. Blood sample (3ml) from each neonate was centrifuged and the serum was stored at −20°C freezer, until the serologic tests were achieved. About 15 ml of urine samples were collected in a sterile container without additives and transported to the laboratory on wet ice. A fresh urine sample was processed for cell culture, and the remaining portion was frozen at −20°C for PCR.

### Viral culture

For processing specimens, a 10-ml volume of urine was centrifuged at 400 × g in a tube containing several glass beads. Approximately 7 ml of the urine supernatant was removed, and the remaining urine and sediment were vortexed for 30 to 60 s. The material was then filtered through a 0.2um-pore-size syringe filter **(Sartorius Stedim- Biotech)** that was first moistened with 1 ml of maintenance medium [Eagle minimal essential medium (EMEM) with 2% fetal calf serum (FCS) containing 200 mM glutamine, 100 U of penicillin per ml, 100 μg of streptomycin per ml and amphotericin B (0.25ug/ml)]. This processed urine specimen, now bacteria free, was used to inoculate tissue culture cells.^10^

MRC-5 cells (10000/ml) were inoculated into tissue TPP 102 tiny tissue culture tubes (MidSci - St. Louis – USA) and were maintained with 4 or 5 ml of EMEM (10% FCS) until a confluent monolayer cells formed. The EMEM was then aspirated and (0.5) ml of specimen was added. The culture tubes were centrifuged at 2,000 × g for 1 h, and the culture fluid was replaced with fresh medium then incubated at 37°C in 5% CO2.^11^ Medium was changed in all culture tubes on day 1 and then every seventh day. 12

CMV was identified by the detection of its characteristic slowly developing focal cytopathic effect.^13^ Detection of CMV was confirmed by immunoflourescent (IF) staining of scraped cells by using a monoclonal antibody reacting with the CMV IE1 and EA gene product (E13 + 2A2; Argene, Varilhes, France). EMEM, FCS and Cell line was supplied as confluent healthy sheet from the Holding Company for Biological Products and Vaccines (VACSERA) Giza-Egypt.

### Case-Control Study

Positive cases by tissue culture and 20 negative cases as a control were investigated for the presence of anti-CMV IgM in blood using ELISA and CMV genome in urine using PCR for comparing both methods using tissue culture as the reference.

### Serology

Anti-CMV IgM was measured by ELISA (CMV Stat M; Diagnostic Automation, Inc.) which was performed according to the manufacturer s instructions.^14^ An enzyme immune-sorbent assay index of 1.0 or greater was considered positive.

### PCR

Urine samples were centrifuged at 1500 rpm for 20 minute. Supernatant was discarded and the pellet was washed with 1X phosphate buffered saline (PBS), three times. The DNA was extracted from the pellet by using the QIAmp viral DNA minikit according to the manufacturer s manual (QIAGEN, Benelux, Netherlands).

Extracted DNA (5 μl) was amplified by a nested PCR with primers specific for the glycoprotein H (UL75) region (outer primer set, 5′-AGG TAT TGA CAG ATC AAT GG-3′ and 5′-CTC CTT CTC TCG GGT GTA AC-3′; inner primer set, 5′-CCA CCT GGA TCA CGC CGC TG-3′ and 5′-TGG TGT TTT CAC GCA GGA A-3′).

PCR reactions for detection of CMV were set up in a clean room with pipettes reserved specifically for this purpose. A master mix was then prepared in an autoclaved 1.5 ml microcentrifuge tube containing 4μl distilled water, 12.5μl Taq PCR Master Mix and 1 μl of forward and reverse primers. The total volume of this master mix was 20μl. It was then briefly vortexed and aliquoted into autoclaved PCR tubes. Then 5 μl of the DNA extract was added to the master mix in each tube, except the negative control tube. Thus, the total volume of the PCR reaction mixture was 25μl. For the second round of amplification, the same was done as the first step except inner primers set were used instead of outer primer set and 5μl of the first round PCR product were used instead of 5μl of the DNA extracted.

**Figure 1:**
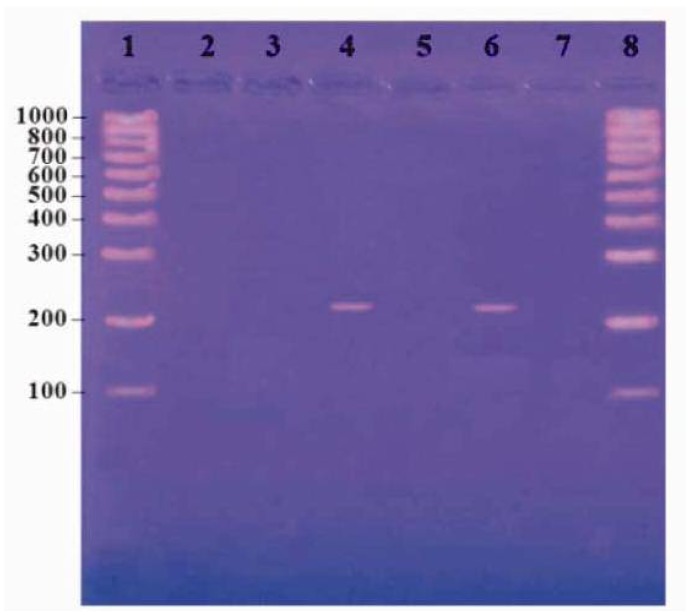
Result of PCR for identification of CMV by agarose gel electrophoresis.

The mixes were overlaid with 2 drops of mineral oil. The conditions were 1 cycle of 10 min at 95°C, followed by 34 cycles of 45 s at 95°C, 1 min at 55°C, and 50 s at 72°C and a final cycle of 10 min at 72°C.^15^ The reaction product was resolved by electrophoresis using 2% agarose gels containing ethidium bromide. A PCR result was considered positive if a DNA band of 215 bp was present. (Figure 1)

### Stastisical analysis

Data are summarized as mean and standard deviation and compared between groups by independent t test. Qualitative data are presented as number and percentages. Association was estimated by chi-square and Fissure exact test was used when expected cell is less than 5. Validity of CMV IgM and PCR is calculated by sensitivity, specificity, positive predictive value, negative predictive value and kappa agreement. Probability is considered significant when less than 0.05.

## Results

In this study 94 suspected neonates were tested for the presence or absence of CMV in urine using tissue culture. Of these 94 cases 6 cases were asymptomatic but born to mothers with a history of primary CMV infection during pregnancy. Three (50%) out of 6 asymptomatic neonates were proved as congenital CMV infection by tissue culture. 88 out of 94 tested cases were neonates with clinical symptoms compatible with congenital CMV. Only 16 (18.2%) out of 88 symptomatic cases were also proved as congenital CMV infection by tissue culture. Only 2 of the symptomatic cases had born to mothers with a history of primary CMV infection during pregnancy.

Nineteen (20.2%) out of 94 suspected neonates were proven positive for congenital CMV infection using viral culture of urine samples. Three out of 19 positive cases were asymptomatic and 16 were symptomatic.

For comparing PCR and ELISA in diagnosis of congenital CMV infection the 19 positive cases proved by tissue culture (CMV group) and 20 negative cases as controls were investigated for the presence of anti-CMV IgM in blood using ELISA and CMV genome in urine using PCR. Tissue culture was the reference for the comparison.

Table 1 shows that the difference between CMV infected group and the control group as regard to sex and age distribution is statistically insignificant (P= 0.42 and 0.9 respectively); on the other hand there is high statistically significant difference between both groups as regard the birth weight, gestational age < 37 and small for gestational age items (P= 0.00, 0.03 and 0.01 respectively).

Table 2 shows that there is statistically insignificant difference as regarding the clinical and laboratory abnormalities detected for neonates of both groups. It also shows that jaundice (63%) and hepato-splenomegaly (42%) were the most common clinical signs in both groups.

Table 3 shows that CMV IgM was detected in 62%, 66% and 25% in SCCMV, ACCMV and control groups respectively. PCR identified CMV in 100%, 100% and 0% of SCCMV, ACCMV and control groups respectively.

Figure 1 shows lane 1 and 8 contain molecular weight marker from 100–1000 bp (**Roche, Lewes, East Sussex).** Lane 2 contains negative control, lane 4 and 6 contain positive cases and lane 3, 5 and 7 contain negative case.

Table 4 shows that 19 out of 19 culture positive samples were also positive by PCR. The sensitivity of PCR was 100%, specificity100%, PPV 100%, NPV 100% and there was excellent agreement between the 2 tests (Kappa coefficient was 1 and P=0.000).

Table 5 shows that 12 positive cases for CMV in urine culture were also positive for IgM CMV. While 7 positive cases for CMV in urine culture were negative for IgM CMV. The sensitivity of IgM CMV was 63.2%, specificity 85%, PPV 80%, NPV 70.8%, and there was good agreement between the 2 tests (Kappa coefficient was 0.48 and P=0.002).

By comparing PCR and IgM CMV ELISA results (using tissue culture as a reference), PCR showed higher sensitivity and specificity than IgM CMV detected by ELISA.

## Discussion

Cytomegalovirus is a beta-herpes virus that leads to congenital infection in 0.4% to 2.3% of all newborns. The risk of intrauterine transmission after primary CMV infection during pregnancy approaches 40%, with an increased risk of adverse fetal effects if infection occurs during the first half of pregnancy. 16 In this study we founded that CMV-infected infants were significantly of low birth weight (2441 ± 250), more likely to exhibit intrauterine growth restriction than the control group (2883 ± 207) and mostly born before 37 weeks of gestational age; this finding is consistent with Morgan et al.,^17^ who reported that CMV infections are of more prevalence in premature and low birth weight neonates in neonatal intensive care units. Also, Mussi-Pinhata et al.^18^ reported that congenital CMV infection resulted in significantly lower mean birth weights. It has been suggested that CMV infects the uterine wall and/or the adjacent placenta, impairing critical aspects of syncytio-trophoblast and cyto-trophoblast differentiation, thus altering their capacity to provide oxygen and nutrients to the developing fetus^19^, In contrast to these results Al-Hareth et al.^6^ reported that low birth weight and small head circumference at birth failed to predict congenital CMV infection.

In this study, jaundice (63%) and hepato-splenomegaly (42%) were the most common clinical signs in both CMV group and control groups. Similarly Ornoy and Diav-Citrin^20^ documented that symptoms of congenital CMV infection include microcephaly, growth retardation, hepato-splenomegaly, chorioretinitis, jaundice, petechiae and hearing impairment.

In this study 19 cases (20.2%) from 94 clinically suspected cases were proven by urine tissue culture to have congenital CMV infection, this is as the results of other investigators in this field who reported near values of 23.6% and 12.57% respectively.^21^,^22^

In this study we use urine samples rather than blood samples to detect CMV infection. PCR of dried blood spot (DBS) has recently been reported by a number of investigators as a useful technique for detecting congenital CMV infection. One limitation to the use of DBS as the sole test for detecting congenital CMV is that the viral load in blood may often be lower than in saliva or urine, or that DNAemia may be absent altogether in a congenitally infected baby. This may make detection of DNA in the DBS relatively insensitive compared to urine or saliva in the diagnosis of congenital CMV.^22^ Urine was found to be the ideal specimen for the detection of CMV as the detection rate in the urine was statistically higher (McNemar test, P < 0.05) than in the blood.^7^

In this work 3 (50%) out of 6 asymptomatic neonates and 16 (18.2%) out of 88 symptomatic neonates were proved as congenital CMV infection by tissue culture. This was in accordance with other investigators who documented that of congenitally infected fetuses only 10% are symptomatic at birth.^22^ that is why there was insignificant difference as regarding the clinical and laboratory abnormalities detected for neonates of both CMV and control groups as shown in table 2.

In this work the 6 asymptomatic and only 2 of the symptomatic cases had born to mothers with a history of primary CMV infection during pregnancy. These values are underestimated as CMV screening is not done as a routine test for pregnant females.

Ocular disease in congenital CMV occurred more frequently (P<0.05) among children who were symptomatic at birth than those who were initially asymptomatic.^23^ In our study the only ocular finding in proven cases of congenital CMV was chorioretinitis which was found in 3 out of 19 cases (15.7%), this is agreed with Jones et al who found that chorioretinitis occurs in 10–15% of symptomatic babies with congenital CMV^24^ and added that microcephaly occurs in around 50% at birth but does not always persist or does not necessarily imply later neurological handicap^24^.

Viral culture of urine has long been the gold standard laboratory test for the diagnosis of CMV infection in newborns. 25 That is why we used it as a reference in comparison between PCR and IgM ELISA in diagnosis of congenital CMV infections.

In this study we found that 19 out of 19 (100%) neonates with positive urine culture for CMV had also positive PCR results and at the same time no one of the control group showed positive PCR result (table 4). On the other hand 12 out of 19 (63%) neonates had positive IgM results and 5 (25%) of the control group had positive IgM results as sown in table (5). This agreed with Christopher et al. 26 who found CMV DNA by PCR of all (100%) newborns with proven or probable congenital CMV infection and detected CMV IgM in only (30%) of these infants.

CMV can be evaluated with viral culture and PCR. Given the time-consuming and costliness of viral culture, PCR has been increasingly utilized to detect CMV infection. 16 Lazzarotto et al. 27 found that the sensitivity and specificity of the PCR for detection of CMV were 98% and 98%, respectively.

This is in accordance with our results where we found that CMV PCR had a sensitivity of 100%, specificity of 100%, positive predictive value of 100%, and negative predictive value of 100%. Our findings about PCR are also with Marianne et al. 21 who found that the ranges of sensitivity, specificity, positive predictive value, and negative predictive value for two different CMV PCR assays were 95.0%–100%; 98.1%–99.0%; 94.1%–96.9%, and 98.5%–100%, respectively.

In this work, by comparing PCR and IgM CMV results (using tissue culture as a reference), PCR for urine sample showed higher sensitivity and specificity than IgM CMV detected by ELISA.

Cytomegalovirus-IgM antibodies assessment in neonatal blood was incapable of detecting all cases of congenital CMV infection in newborns. This might be due to the undetectable amount of CMV-IgM antibodies in blood by the method used, the insensitivity of CMV-IgM measurement in detecting congenital CMV infection, and/or delayed development of CMV-IgM in infected fetuses.^6^

In the evaluation of a newborn infant with possible congenital CMV, care must be taken not to rely on antibody titers in the infant (so-called TORCH titers) because these are seldom of value in establishing the diagnosis of congenital CMV. The finding of CMV antibodies in an infant may simply reflect transplacental transfer of IgG, and IgM may not be detected in all cases.^25^ The most important diagnostic studies in the evaluation of suspected CMV disease are virologic studies, not serologic studies, including viral culture and PCR.^27^

Early diagnosis of congenital CMV infection is essential in order to start preemptive treatments and reduce consequent sequelae such as deafness and heart malformation.^6^ Also trials regarding anti-viral treatment of the symptomatic babies with ganciclovir have shown that the infected babies show positive response to the drug.^28^

## Conclusions

This study illustrates the potential role of urine PCR as an important diagnostic method for congenital CMV infection, and refers to the lack of sensitivity of the newborn IgM status for the same purpose. This study concluded that PCR using urine sample is more sensitive and specific technique for detection of congenital CMV infection than CMV IgM. Being more cost effective, less cumbersome and less time consuming in relation to viral culture, PCR may be used in detection of congenital CMV infection in suspected neonates.

## Figures and Tables

**Table 1:** Demographic data of the neonates in the CMV group and control groups.

*Data*		*CMV group (n=19)*	*Control group (n=20)*	*P value*
***Sex***	♂	11	9	0.42
	♀	8	11	
***age***	*1^st^week*	6	7	0.90
	*2^nd^week*	5	6	
	*3^rd^week*	8	7	
***Birth weight(g)***		2441 ± 250	2883 ± 207	0.00
***Gestational age < 37(w)***		10 (52.6%)	4 (20%)	0.03
***Small for gestational age***		11(57.9%)	4(20%)	0.01

**Table 2:** Clinical and laboratory abnormalities of both CMV and negative control groups.

		CMV group *(n=19)*	Control group *(n=20)*	P value
**Clinical Signs**	*Microcephaly*	*2 (10.5%)*	0 (0%)	0.23
	*Seizures*	*3 (15.7%)*	1 (5%)	0.34
	*Hepato-splenomegaly*	*8 (42%)*	5 (25%)	0.27
	*Purpura*	*3(15.7%)*	2 (10%)	0.62
	*Petechiae*	*3 (15.7%)*	4 (20%)	0.69
	*Jaundice*	*12 (63%)*	9 (45%)	0.43
	*Chorio-retinitis*	*3(15.7%)*	0 (0%)	0.29
**No. of signs**	*1*	*6 (31.5%)*	7 (35%)	0.65
	*2*	*4 (21%)*	3 (15%)	0.69
	≥ *3*	*6 (31.5%)*	2 (10%)	0.34
**Lab. findings**	*Thrombocytopenia*	*4 (21%)*	2 (10%)	0.23
	↑ *alanine aminotransferase*	*4 (21%)*	1 (5%)	0.35
	↑ *aspartate aminotransferase*	*6 (31.5%)*	3 (15%)	0.71

**Table 3:** CMV IgM and PCR results for symptomatic congenital CMV (SCCMV), asymptomatic congenital CMV (ACCMV) and control groups.

*TEST*	*SCCMV (n=16)*	*ACCMV (n=3)*	Control group *(n=20)*
***CMV IgM***	10/16 (62%)	2/3 (66%)	5 (25%)
***PCR***	16/16 (100%)	3/3 (100%)	0 (0%)

**Table 4:** Comparison between PCR-based detection assay and tissue culture for the identification of congenital CMV.

		Culture result		
		*Positive*	*Negative*	*Total*
**PCR results**	***Positive***	19	0	19
	***Negative***	0	20	20
	***Total***	19	20	39

Sensitivity =100%, Specificity = 100%, Positive predictive value = 100%, Negative predictive value = 100%, Kappa = 1 and P value = 0.0000.

**Table 5:** Comparison between IgM CMV detection assay and tissue culture for the identification of CMV.

		Culture result		
		*Positive*	*Negative*	*Total*
**IgM CMV**	***Positive***	12	3	15
	***Negative***	7	17	24
	***Total***	19	20	39

Sensitivity = 63.2%, Specificity = 85%, Positive predictive value = 80%, Negative predictive value = 70.8%, Kappa = 0.48 and P value = 0.002.
